# Pharmacotherapy of the Lipid-Lowering Drugs: Update on Efficacy and Risk

**DOI:** 10.3390/ijms24020996

**Published:** 2023-01-04

**Authors:** Sabata Pierno, Olimpia Musumeci

**Affiliations:** 1 Section of Pharmacology, Department of Pharmacy and Drug Sciences, University of Bari “Aldo Moro”, 70125 Bari, Italy; 2 Unit of Neurology and Neuromuscular Disorders, Department of Clinical and Experimental Medicine, University of Messina, 98125 Messina, Italy

Lipid-lowering drugs are widely used for the prevention and cure of cardiovascular diseases (CVD). Different drugs are used in therapy. Statins, or 3-hydroxy-3-methylglutaryl-coenzyme-A reductase inhibitors, are among the most-used drugs worldwide. These drugs are able to lower blood LDL-cholesterol (LDL-c) levels and to hamper atherosclerosis. However, it should be considered that they can cause undesired side effects. Thus, this Special Issue was aimed to update on the benefit/risk ratio (Figure 1). Statin is associated with intolerance, and in particular, muscle symptoms (statin-associated muscle symptoms-SAMS), ranging from isolated muscle pain (myalgia) to (rarely) muscle weakness and sustained high CK levels (myopathy) that may lead to poor adherence to therapy, resulting in a failure to achieve the desired cholesterol reduction [1]. The case of cerivastatin is well known because it was withdrawn from the market because of an increased risk of rhabdomyolysis, which caused renal failure and death [2]. Although new drugs are emerging (monoclonal antibodies, antisense oligonucleotides, etc.), statins remain the first-line option for the treatment of hypercholesterolemia, making it essential to identify the causes of their toxicity.

The review of Camerino et al. presents an update at 2021 [3] on the risk of myopathy and describes new biomarkers of muscle toxicity. These drugs may affect gene transcription and ion transport, as observed in preclinical and clinical studies, contributing to muscle function impairment. It has been shown that sarcolemma ClC-1 chloride channel protein is reduced in patients with SAMS, and this is often associated with alterations of the electromyographic recordings and myotoxicity occurring as muscle necrosis and manifesting with increased CK levels. ClC-1 plays an important role in sarcolemma excitability and contractility [4,5,6] and its reduction in skeletal muscles of patients using statins may help to evaluate the risk of myopathy [7,8]. Importantly, this review also focused on the physiological and pathological situations in which statin therapy should be avoided for the presence of aggravating factors. Mainly, gender, age, genetic variants or polymorphisms of metabolizing enzymes and/or of transporters, drug interaction, alcohol abuse, hypothyroidism, extreme physical exercise, and serum Vitamin D deficiency are predisposing factors for SAMS. The adverse events associated with statins may also be strengthened in patients presenting comorbidities such as Myotonia Congenita, Huntington’s disease, Amyotrophic Lateral Sclerosis, Mitochondrial Myopathy, and Diabetes. Thus, statin treatment needs to be personalized. The European Atherosclerosis Society (EAS) Consensus Panel described the pathophysiology of SAMS and provided a guideline for diagnosis and management of the risk at the aim to ensure the right therapy [9]. Alternatively, other drugs can be prescribed, such as bile acid sequestrants, ezetimibe, colesevelam, lomitapide and also nutraceutical compounds to substitute statin therapy [10,11,12]. The bempedoic acid is a small molecule that inhibits ATP-cytrate lyase, a cytosolic enzyme upstream of HMGCoA reductase in the pathway of cholesterol synthesis. This drug seems to show less muscle-related side effects [13,14].

A novel target for lipid-lowering therapy is the proprotein convertase subtilisin/kexin type 9 (PCSK9). The inhibitors of PCSK9 have been recently drawn as powerful drugs for lowering LDL-c. PCSK9 is a protein that promotes the degradation of hepatic LDL receptors (LDL-R). Thus, the monoclonal antibodies (evolocumab, alirocumab) are effective in the inhibition of PCSK9 activity [15]. Moreover, inclisiran is a small interfering RNA targeting the mRNA of PCSK9 specifically in the liver. These drugs are found to be highly effective, more than statins, and may offer a long-duration effect. At this regard, the review of Basiak et al. 2021 [16] describes the mechanism of the PCSK9 inhibitors (alirocumab, evolocumab, and the discontinued bocucizumab) in the binding to PCSK9 and in the reduction of LDL-R degradation and, hence, in the increase of the LDL-c uptake from the bloodstream. In line with the American Heart Association (AHA) and the European Society of Cardiology (ESC) guidelines, these drugs are indicated in case of familial hypercholesterolemia and in cases of statin intolerance or in those individuals at very high risk and with persistent high risk despite being treated with a maximally tolerated statin, or in combination with ezetimibe. In monotherapy and/or in combination with statins and/or ezetimibe, they are useful to intensify therapy and achieve therapeutic goals. The authors also collected and reported all the available literature describing the pleiotropic effects of these drugs, such as the anti-atherosclerotic effect, the anti-aggregation effect, the anti-coagulant effect, the anti-neoplastic effect, and the ability to influence the bacterial infections. In particular, in dyslipidemic patients, PCSK9 inhibitors decreased the level of the biochemical markers of atherosclerotic plaque progression. An important advantage of using PCSK9 inhibitors is their better tolerance if compared with statins. This is a new class of drugs that still require confirmatory studies, but if confirmed they can replace statin therapy when needed.

The review of Attardo et al. 2022 [17] focused on statin-associated neuromuscular adverse effects and also on diagnosis and management. Indeed, the authors, despite the more known SAMS, described the possible occurrence of peripheral neuropathy with the use of statins and the dangerous ability of these drugs to unmask pre-existing neuromuscular junction dysfunction. The authors claim that these effects represent about two-thirds of all adverse events. At this aim, is important that a clinical follow up of patients be conducted, assuming statins to reveal early side effects that may cause neuromuscular damage, but also evaluating the risk before starting statins. Additionally, pharmacogenomic studies as well as patient lifestyle will help to timely predict neuromuscular complications due to statin exposure. This is also important to plan how to proceed, whether suspending and substituting statins or decreasing the dose to avoid adverse events. It should be considered that patients with neuromuscular disorders and genetic myopathies, such as Pompe disease, Kennedy disease, McArdle disease, myotonic dystrophy 1 and 2, and muscle phosphorylase b kinase deficiency may be more predisposed to develop SAMS. Genetically speaking, it has been identified that some gene mutations are related with SAMS phenotypes such as *COQ2, HTR7, RYR1, GATM, CYP3A4, CYP2D6, ABCC2, RYR2, CLCN1, VDR,* and *ABCG2*. It was found that also the LPIN1 gene mutation may lead to myopathy and rhabdomyolysis in children [18]. Among neuromuscular manifestations of statin intolerance, muscle symptoms (myopathy, myalgia, cramps) are the more frequent, rarer, peripheral neuropathies may occur with axonal involvement and nerve conduction damage. Another important condition reported is an immune-mediated necrotizing myopathy (IMNM) characterized by autoantibodies directed against 3-hydroxy3-methyl-glutaryl-coenzyme-A reductase (HMGCR). This condition is a severe autoimmune myopathy, which does not improve with statin withdrawal but needs specific immunosuppressive therapy. There is a strong association between IMNM in patients exposed to statins and anti-HMGCR antibodies: statins increase HMGCR expression in muscle and other tissues and antibody levels correlate with the severity of myopathy [19]. The lack of symptoms resolution after discontinuation of statins may suggest an IMNM condition.

The important contribution of Macchi et al. 2022 [20] underlined that only a small part of atherosclerotic cardiovascular disease (ASCVD) high-risk patients remains adherent to statin therapy after 5 years, possibly due to discontinuation related to intolerance and non-adherence, two of the major gaps in both primary and secondary prevention. Indeed, statins are often used in chronic therapy and the discontinuation of therapy may increase the risk of cardiovascular disease [21]. For this reason, the authors would like to understand the underlying mechanisms of SAMS at the aim to improve the therapeutic approach and support adherence in patients needing to be treated. They evaluated the impact of high doses of atorvastatin on skeletal muscle mitochondrial activity and axonal excitability in a murine model of atherosclerosis (*ApoE^-/-^* mice fed with a high fat, high cholesterol diet). Atorvastatin induced a reduction of mitochondrial basal respiration and of ATP production in skeletal muscles. Moreover, atorvastatin altered the responsiveness of mechanoceptive and nociceptive fibres. These findings point out to a mild sensitization on mechanical, tactile, and pain sensitivity in accord with axonal neuropathy. The authors suggested that this effect may be consequent to an alteration in axonal structure and secondary to changes in lipid membrane composition. Overall, these results demonstrated a peripheral neuropathy accompanied by impaired energy homeostasis and suggest a guide for drug intervention or lifestyle changes to prevent statin-induced muscle symptoms and to maintain statin therapy for long time period.

Important findings are reported in the paper of Stephens et al. 2021 [22] which described the effect of statins on lymphatic vascular smooth muscle cell function, a role that has never been investigated before. Lovastatin and simvastatin showed to increase vessel contraction frequency in a concentration-dependent manner, an effect that culminates with a complete loss of tone and with the inability of the vessels to achieve lymph propulsion. This effect likely depends on the RhoA down-regulation and on the Rho-associated protein Kinase (ROCK) decreased activation (due to the statin-induced inhibition of HMGCR activity), which is able to phosphorylate the myosin light chains and to control vessel contraction. This phenomenon was found to be restricted to drugs in the lactone-ring form and independent of NO or prostaglandin. It is known that the lymphatic system participates directly in the increased uptake of lipophilic molecules. The authors also describe that acute in vivo treatment of rats with lovastatin, but not lovastatin-hydroxyacid, resulted in a significantly decreased dietary lipid absorption through decreased lymphatic contractility and loss in lymphatic vessel ejection fraction. Their findings reveal a novel mechanism by which statins actively lower circulating cholesterol by decreasing intestinal lymphatic lipid absorption, transport, and peripheral dissemination of dietary absorbed lipids.

The role of natural compounds in the reduction of lipid profile during metabolic syndrome and/or obesity was also investigated. The paper of Shipelin et al. 2021 [23] described an hypolipidaemic role for two amino acids such as tyrosine (Tyr) and tryptophan (Trp) that showed to play a significant role in the regulation of lipid and energy metabolism, locomotor activity, and eating behavior. The authors have studied the possibility to modulate these processes by increasing the pool of Tyr and Trp in the diet. However, the two amino acids showed different activity. Trp led to a normalization of the body weight in a rat model of obesity almost to the control level. It seems that this effect is a consequence of increased energy expenditure due to the raised locomotor activity in these animals. The Tyr supplementation led to the normalization of triglycerides and LDL. The liver tissue morphology showed that the consumption of Tyr weakened the signs of fatty degeneration. Tyr was characterized by the ability to prevent excessive lipid accumulation in the liver. The addition of Trp indeed led to an unfavorable effect, consisting of the appearance of a high number of fatty vacuoles and lipid accumulation. The data obtained indicate a more pronounced anti-inflammatory effect of Tyr as compared to Trp, in terms of suppression of the production in circulating levels of cytokines (interleukines IL-1, IL-6, and IL-17A) as occurs during development of diet-induced obesity and metabolic syndrome. Tyr influences energy metabolism in the muscles by the intensification fatty acids beta-oxidation as indicated by the increase in the acetyl-CoA. In conclusion, the authors show the possibility to modulate energy metabolism, physical activity, and eating behavior by changing the ratio of Trp and Tyr supplied with food in the pool of dietary amino acids.

In conclusion, in this Special Issue reported significant advances in the field of lipid-lowering therapy efficacy and risk factors. Several aspects have been examined both at the preclinical and clinical levels for a better use of statins and/or of biological drugs, as well as of natural compounds, to make the right choice for a personalized therapy.

## Figures and Tables

**Figure 1:**
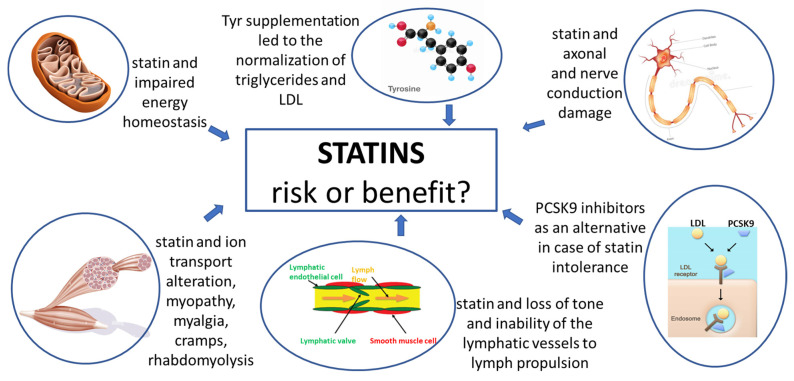
This figure summarized the take-home message contained in the papers published in the Special Issue “Pharmacotherapy of the Lipid-Lowering Drugs: Update on Efficacy and Risk” aimed to update on the benefit/risk ratio of statin and other lipid-lowering drugs. Different causes of risk are detailed as well as drugs and natural compounds effective in the therapy of hyperlipidemia.

## Data Availability

Not applicable.

## References

[1] Ward N.C., Watts G.F., Eckel R.H. (2019). Statin Toxicity. Circ Res..

[2] Staffa J.A., Chang J., Green L. (2002). Cerivastatin and reports of fatal rhabdomyolysis. New Engl. J. Med..

[3] Camerino G.M., Tarantino N., Canfora I., De Bellis M., Musumeci O., Pierno S. (2021). Statin-Induced Myopathy: Translational Studies from Preclinical to Clinical Evidence. Int. J. Mol. Sci..

[4] De Luca A., Pierno S., Camerino D.C. (1997). Electrical properties of diaphragm and EDL muscles during the life of dystrophic mice. Am. J. Physiol. Cell Physiol..

[5] Capogrosso R.F., Mantuano P., Uaesoontrachoon K., Cozzoli A., Giustino A., Dow T., Srinivassane S., Filipovic M., Bell C., Vandermeulen J. (2018). Ryanodine channel complex stabilizer compound S48168/ARM210 as a disease modifier in dystrophin-deficient mdx mice: Proof-of-concept study and independent validation of efficacy. FASEB J..

[6] Cozzoli A., Liantonio A., Conte E., Cannone M., Massari A.M., Giustino A., Scaramuzzi A., Pierno S., Mantuano P., Capogrosso R.F. (2014). Angiotensin II modulates mouse skeletal muscle resting conductance to chloride and potassium ions and calcium homeostasis via the AT1 receptor and NADPH oxidase. Am. J. Physiol. Cell Physiol..

[7] Camerino G.M., Musumeci O., Conte E., Musaraj K., Fonzino A., Barca E., Marino M., Rodolico C., Tricarico D., Camerino C. (2017). Risk of Myopathy in Patients in Therapy with Statins: Identification of Biological Markers in a Pilot Study. Front. Pharmacol..

[8] Camerino G.M., De Bellis M., Conte E., Liantonio A., Musaraj K., Cannone M., Fonzino A., Giustino A., De Luca A., Romano R. (2016). Statin-induced myotoxicity is exacerbated by aging: A biophysical and molecular biology study in rats treated with atorvastatin. Toxicol. Appl. Pharmacol..

[9] Stroes E.S., Thompson P.D., Corsini A., Vladutiu G.D., Raal F.J., Ray K.K., Roden M., Stein E., Tokgözoglu L., Nordestgaard B.G. (2015). Statin-associated muscle symptoms: Impact on statin therapy—European Atherosclerosis Society Consensus Panel Statement on Assessment, Aetiology and Management. Eur. Heart J..

[10] Nikolic D., Banach M., Chianetta R., Luzzu L.M., Stoian A.P., Diaconu C.C., Citarrella R., Montalto G., Rizzo M. (2020). An overview of statin-induced myopathy and perspectives for the future. Expert Opin. Drug Saf..

[11] Norata G.D., Tibolla G., Catapano A.L. (2014). Targeting PCSK9 for hypercholesterolemia. Annu. Rev. Pharmacol. Toxicol..

[12] Russell C., Sheth S., Jacoby D. (2018). A Clinical Guide to Combination Lipid-Lowering Therapy. Curr. Atheroscler. Rep..

[13] Rosenson R.S., Baker S., Banach M., Borow K.M., Braun L.T., Bruckert E., Brunham L.R., Catapano A.L., Elam M.B., Mancini G.B.J. (2017). Optimizing Cholesterol Treatment in Patients With Muscle Complaints. J. Am. Coll. Cardiol..

[14] Ruscica M., Banach M., Sahebkar A., Corsini A., Sirtori C.R. (2019). ETC-1002 (Bempedoic acid) for the management of hyper-lipidemia: From preclinical studies to phase 3 trials. Expert Opin. Pharmacother..

[15] Lloyd-Jones D., Morris P., Ballantyne C.M., Birtcher K.K., Covington A.M., DePalma S.M., Minissian M.B., Orringer C.E., Smith S.C., Waring A.A. (2022). ACC Expert Consensus Decision Pathway on the Role of Nonstatin Therapies for LDL-Cholesterol Lowering in the Management of Atherosclerotic Cardiovascular Disease Risk. J. Am. Coll. Cardiol..

[16] Basiak M., Kosowski M., Cyrnek M., Bułdak Ł., Maligłówka M., Machnik G., Okopień B. (2021). Pleiotropic Effects of PCSK-9 Inhibitors. Int. J. Mol. Sci..

[17] Attardo S., Musumeci O., Velardo D., Toscano A. (2022). Statins Neuromuscular Adverse Effects. Int. J. Mol. Sci..

[18] Brunham L.R., Baker S., Mammen A., Mancini G., Rosenson R.S. (2018). Role of genetics in the prediction of statin-associated muscle symptoms and optimization of statin use and adherence. Cardiovasc. Res..

[19] Mohassel P., Mammen A.L. (2018). Anti-HMGCR Myopathy. J. Neuromuscul. Dis..

[20] Macchi C., Bonalume V., Greco M.F., Mozzo M., Melfi V., Sirtori C.R., Magnaghi V., Corsini A., Ruscica M. (2022). Impact of Atorvastatin on Skeletal Muscle Mitochondrial Activity, Locomotion and Axonal Excitability—Evidence from ApoE-/- Mice. Int. J. Mol. Sci..

[21] Ruscica M., Ferri N., Banach M., Sirtori C.R., Corsini A. (2022). Side effects of statins: From pathophysiology and epidemiology to diagnostic and therapeutic implications. Cardiovasc. Res..

[22] Stephens M., Roizes S., von der Weid P.-Y. (2021). Off-Target Effect of Lovastatin Disrupts Dietary Lipid Uptake and Dissemination through Pro-Drug Inhibition of the Mesenteric Lymphatic Smooth Muscle Cell Contractile Apparatus. Int. J. Mol. Sci..

[23] Shipelin V.A., Trusov N.V., Apryatin S.A., Shumakova A.A., Balakina A.S., Riger N.A., Gmoshinski I.V., Nikityuk D.B. (2021). Effects of Tyrosine and Tryptophan in Rats with Diet-Induced Obesity. Int. J. Mol. Sci..

